# Cryo-EM structures of African swine fever virus topoisomerase

**DOI:** 10.1128/mbio.01228-23

**Published:** 2023-08-23

**Authors:** Yan Zhao, Wenhua Kuang, Qiyin An, Jinyue Li, Yong Wang, Zengqin Deng

**Affiliations:** 1 Wuhan Institute of Virology, Center for Antiviral Research, Chinese Academy of Sciences, Wuhan, Hubei, China; 2 University of Chinese Academy of Sciences, Beijing, China; 3 Hubei Jiangxia Laboratory, Wuhan, Hubei, China; Case Western Reserve University School of Medicine, Cleveland, Ohio, USA

**Keywords:** African swine fever virus, cryo-EM, topoisomerase, P1192R, drug target

## Abstract

Type II topoisomerases ubiquitously exist in cellular organisms, where they play an essential role in resolving the topological problems of DNA. The viral type II topoisomerase encoded by the African swine fever virus (ASFV) is critical for viral replication and infection, thus representing an attractive target for antiviral drug development. Here we report two cryo-EM structures of ASFV topoisomerase P1192R in distinct conformations at an overall resolution of 3.16 Å and 3.13 Å, respectively. P1192R assembles as a homodimer with the C-gate formed by the coiled-coil domain adopting a closed or open conformation before reaction, providing the first visual evidence for the dynamic motions of the C-gate of type II topoisomerase. Comparative structural comparisons of eukaryotic homologs and P1192R reveal the unique structural features of P1192R, including the active site configuration, a flexible loop in the TOPRIM domain, an additionally inserted α-helix in the tower domain, and a pin-like structure in the C-terminal coiled-coil domain, which are important for enzyme activity and protein folding. These findings provide important insights into the structure and function of viral topoisomerases and may guide the efficient development of anti-ASFV drugs. Moreover, our study also offers structural evidence to support the scenario of the viral origin of eukaryotic type IIA topoisomerases.

**IMPORTANCE:**

African swine fever virus (ASFV) is a highly contagious virus that causes lethal hemorrhagic diseases known as African swine fever (ASF) with a case fatality rate of 100%. There is an urgent need to develop anti-ASFV drugs. We determine the first high-resolution structures of viral topoisomerase ASFV P1192R in both the closed and open C-gate forms. P1192R shows a similar overall architecture with eukaryotic and prokaryotic type II topoisomerases, which have been successful targets of many antimicrobials and anticancer drugs, with the most similarity to yeast topo II. P1192R also exhibits differences in the details of active site configuration, which are important to enzyme activity. These two structures offer useful structural information for antiviral drug design and provide structural evidence to support that eukaryotic type IIA topoisomerase likely originated from horizontal gene transfer from the virus.

## INTRODUCTION

Topological problems, including intertwining, knotting, and catenation of DNA molecules, occur in many essential biological processes, such as genome replication, transcription, recombination, and chromosome segregation (1). Living organisms evolve a class of proteins known as topoisomerases, which can regulate the topology of DNA by cleaving the phosphodiester bonds, reassigning the spatial locations of DNA strands, and resealing the DNA breaks. According to the strand scission activity, topoisomerases could be classified into two major types: type I and type II, creating single- and double-stranded breaks in DNA, respectively (2 – 4). Topoisomerases universally exist in three domains of life, namely, Archaea, Bacteria, and Eukarya. It is now discovered that some bacteriophages and nucleocytoplasmic large DNA viruses (NCLDVs), including poxviruses, mimiviruses, iridoviruses, and African swine fever virus (ASFV), also encode their own topoisomerases to assist viral replication and infection (5 – 9).

Type II topoisomerases are the most widespread topoisomerases and are further divided into type IIA and IIB based on their sequence and structure. Type IIA enzymes include topoisomerase II (topo II) encoded by both eukaryotes and viruses, and DNA gyrase and topoisomerase IV (topo IV) encoded by bacteria. Type IIB contains a single type of enzyme, topoisomerase VI (topo VI), only found in archaea and plants (10, 11). Despite the low-sequence homology, almost all type II topoisomerases possess three functional domains, namely, an N-terminal ATPase domain with ATP binding site, a central DNA binding and cleavage domain with conserved active site tyrosine residue, and a variable C-terminal domain, which together assemble into homo- or heterodimers (12, 13). Type II topoisomerases can relax supercoiled DNA by a concerted strand-passage mechanism in which the enzyme first cleaves one DNA duplex (G-segment) and then guides the second DNA duplex (T-segment) to pass through the transient breaks (14). During the process of G-segment cleavage, the active site tyrosine forms a transient phosphodiester bond with the end of nicked stands to avoid recombination or rearrangement of the broken DNA. In addition, type II topoisomerases can regulate the association and dissociation of the dimer interfaces (or gates) by conformational changes of individual domains to allow the T-segment transport and release (15 – 21). Since the temporary enzyme-mediated DNA breaks have potentially cytotoxic effects, type II topoisomerase is an ideal drug target for antibacterial and anticancer treatments (22 – 24). Currently, various small molecular drugs targeting type II topoisomerases have been in use, such as quinolones (antibacterial agents) and etoposide (anticancer drug), both trapping the DNA-enzyme complex by inhibiting the resealing of broken strands to produce more DNA lesions that cause cell damage and death (25, 26).

ASFV is a large double-stranded DNA virus belonging to the genus *Asfivirus* in the family *Asfarviridae,* order *Asfuvirales*. It is a highly contagious virus that causes lethal hemorrhagic diseases known as African swine fever (ASF) with a case fatality rate of 100% (27, 28). The ASF epidemic first occurred in Africa and spread rapidly to other countries, resulting in enormous economic losses to the pig industry and has been recognized as the most devastating viral disease of both domestic and feral swine. However, no approved vaccines or specific therapeutic drugs are currently available for treating ASF. ASFV has a large genome containing 150–167 open reading frames and encodes multiple enzymes required for viral transcription and replication (29). Among these genes, the *p1192r* gene specifically encodes viral type II topoisomerase, which is highly conserved in different ASFV isolates. Previous studies showed that transcription of the *p1192r* gene begins at 2 h post-ASFV infection and is active during the entire course of infection. The siRNAs targeting *p1192r* can inhibit viral gene transcription and impair the production of viral factories in infected cells (9, 30, 31). These findings indicate that P1192R plays a crucial role in viral DNA genome replication and expression and provides a promising antiviral target against ASFV.

Structures and functions of eukaryotic and prokaryotic topoisomerases have been extensively studied, which greatly enriched our understanding of the catalytic mechanisms for this important enzyme, and drugs targeting eukaryotic and prokaryotic topoisomerases have been successfully developed. However, mechanistic understandings of viral topoisomerases and antiviral drug development are largely limited, partially due to a lack of high-resolution structural information on viral topoisomerases. In this work, we present two cryo-EM structures of ASFV P1192R in different conformations, revealing a conserved overall architecture similar to homologs from eukaryotes and prokaryotes. Nevertheless, P1192R displays some degrees of structural divergences in the tertiary and quaternary architectures, which may be closely related to its enzyme activity. Our work characterizes the structural features and functional properties of the ASFV P1192R, providing a basis for understanding the mechanism of viral topoisomerases and benefiting the development of anti-ASFV drugs targeting P1192R. Moreover, the P1192R structures presented here also have important implications for understanding the evolution of type II topoisomerase.

## RESULTS

### Structure determination of ASFV P1192R

**Fig 1:**
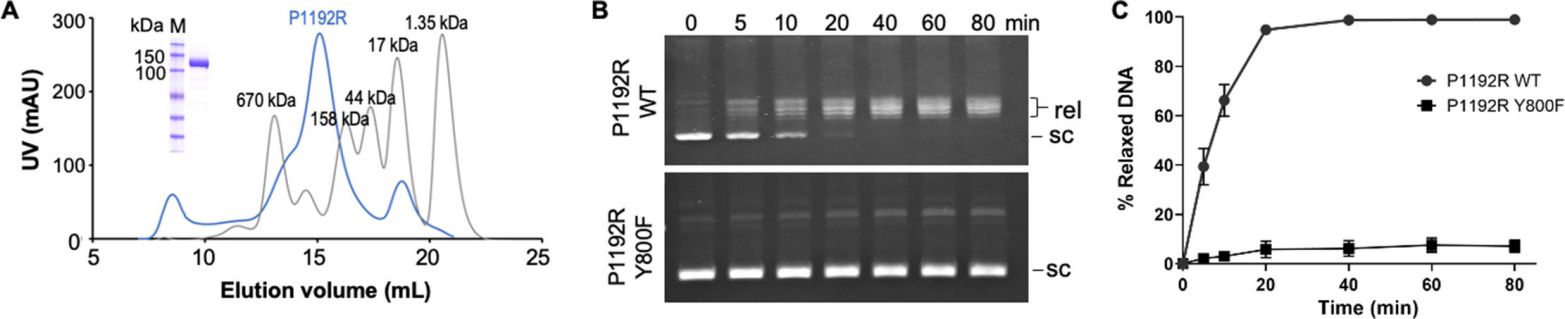
Purification and relaxation activities of ASFV P1192R. (**A**) Representative size-exclusion chromatography and Coomassie-stained denaturing gel show the high monodispersity and purity of the P1192R protein. P1192R was purified using a Superose 6 Increase 10/300 Gl column. The gel filtration profiles of P1192R and standard proteins are colored in blue and gray, respectively. (**B**) and (**C**) time course of relaxation activity of WT P1192R and catalytic tyrosine mutant Y800F, respectively. The pUC19 plasmids were incubated with 30 nM WT or mutant protein at 30°C. The reaction time is indicated above each lane. Supercoiled (sc) and relaxed (rel) topoisomers are indicated. Data are presented as mean values ± SD for three independent experiments.

For structural analyses, we expressed the full-length P1192R in the yeast *Pichia pastoris* and purified the protein using nickel affinity chromatography followed by size-exclusion chromatography. The nickel affinity-purified protein was eluted from a Superose 6 Increase 10/300 Gl column as a monodispersed peak (Fig. 1A). In the DNA relaxation assay using a negatively supercoiled pUC19 plasmid as the substrate, purified P1192R could relax the supercoiled plasmids into relaxed forms within 5 min. The majority of the substrates were converted into products after about 20 min, whereas the catalytic tyrosine mutant (Y800F) almost lost the relaxation activity under the same reaction conditions (Fig. 1B and C). These results indicate that the recombinant P1192R produced by *Pichia pastoris* is a functional topoisomerase, consistent with previous study that P1192R expressed by the yeast *Saccharomyces cerevisiae* is active (9).

To characterize the structural features of type II topoisomerase encoded by the virus, we applied the full-length WT ASFV P1192R protein to single-particle cryo-EM analysis. Interestingly, 3D classification revealed two distinct classes with almost even particle populations, generating high-resolution reconstructions with C2 symmetry imposed at an overall resolution of 3.16 Å and 3.13 Å, respectively (Fig. S1). The high-quality maps allowed us to solve the P1192R structures in two distinct dimeric conformations. One state has a closed conformation in the C-terminal dimer interface, and the other one appears open (Fig. S1). The N-terminal ATPase domain comprising amino acids 1–414 was invisible in both states, probably owing to its flexible connection with the other part of P1192R. Besides the ATPase domain, the cryo-EM densities corresponding to the region covering residues 471–501 also could not be traced in these two states. The final atomic models have good stereochemistry and fit well into the density maps (Table S1).

### Overall architecture of P1192R

**Fig 2:**
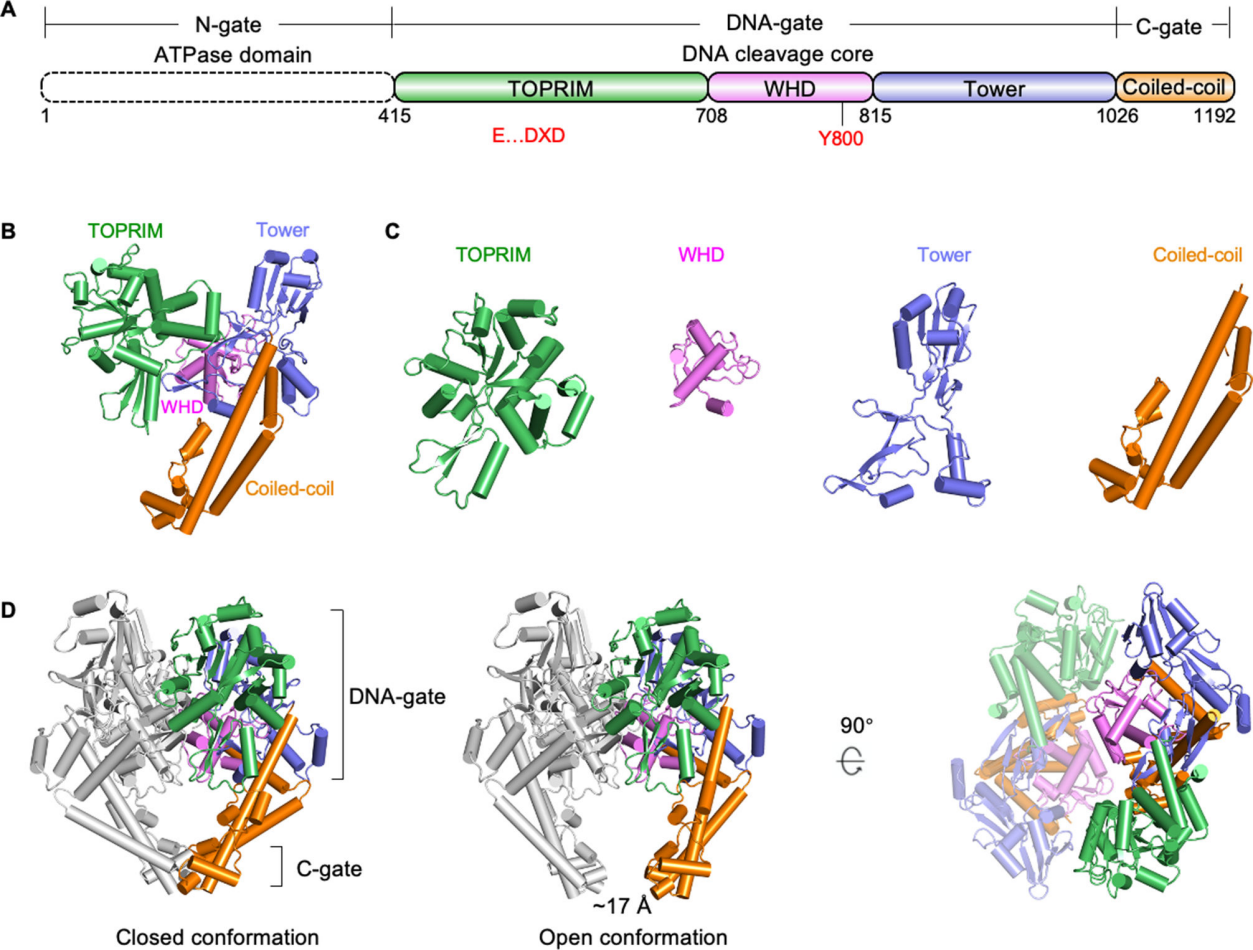
Overall structure of ASFV P1192R. (**A**) Linear domain organization of ASFV P1192R protein. Functional domains, dimerization gates, and important residues are labeled and highlighted. The active site residues including catalytic tyrosine (Y800) and metal-binding residues (E…DXD) are colored in red. (**B**) and (**C**) Cartoon representations of P1192R monomer and individual domains. Domains are colored as in (**A**). (**D**) P1192R dimer structures with their C-gates in closed or open state. One protomer is colored as in (**A**), and the other is shaded gray. The coiled-coil arm is separated by a 17 Å gap in the open state. The right panel shows the dimer interface formed by the DNA cleavage core of P1192R with one protomer shown as semi-transparent.

ASFV P1192R contains two conserved domains, namely, an N-terminal ATPase domain and a central DNA cleavage core followed by a coiled-coil arm, but lacks the nonconserved C-terminal domain (CTD) with modulating activities reported in human topo II (32). The DNA cleavage core was further divided into three small subdomains, termed the topoisomerase-primase (TOPRIM) domain, the winged-helix domain (WHD), and the tower domain (Fig. 2A). The P1192R monomer folds a clamp-like global structure, with the TOPRIM and tower domains lying at opposite constitute the two clips, the coiled-coil serves as the handle, and the WHD domain is wrapped in the center by other domains (Fig. 2B). The TOPRIM domain of P1192R consists of a central nine β-sheets surrounded by several helices with different lengths and harbors a conserved E…DXD motif responsible for metal-ion binding (Fig. 2C). The WHD domain is a five-helix bundle bearing the strictly conserved catalytic tyrosine. The tower domain has a compact “spire” containing two antiparallel β-strands packed against a four-helix bundle and a loose “base” consisting of mixed structural elements. The coiled-coil domain is characterized by three long α-helical lever arm structures flanked with an insertion formed by six short helices (Fig. 2C).

Similar to previously reported topo II structures of yeast and human, P1192R assembles into a large homodimer mediated by two interfaces (Fig. 2D). One is formed by contacts of the subdomains of the DNA cleavage core in each monomer, and the other occurs between the C-terminal coiled-coil arms. These two dimer interfaces constitute the respective DNA-gate and C-gate essential for DNA transport. In our study, two distinct P1192R dimeric states were observed simultaneously in the cryo-EM samples, with their DNA-gate closed, whereas their C-gate is closed and open, respectively. In the open state structure, the interface of coiled-coil arms separates by ~17 Å, comparable with reported C-gate opening structures (17, 18), thereby displaying an opening C-gate conformation (Fig. 2D). These structural data allow us to trace the dynamic changes of C-gate close to physiological state for the first time.

### Conformational changes in the C-gate of P1192R

**Fig 3:**
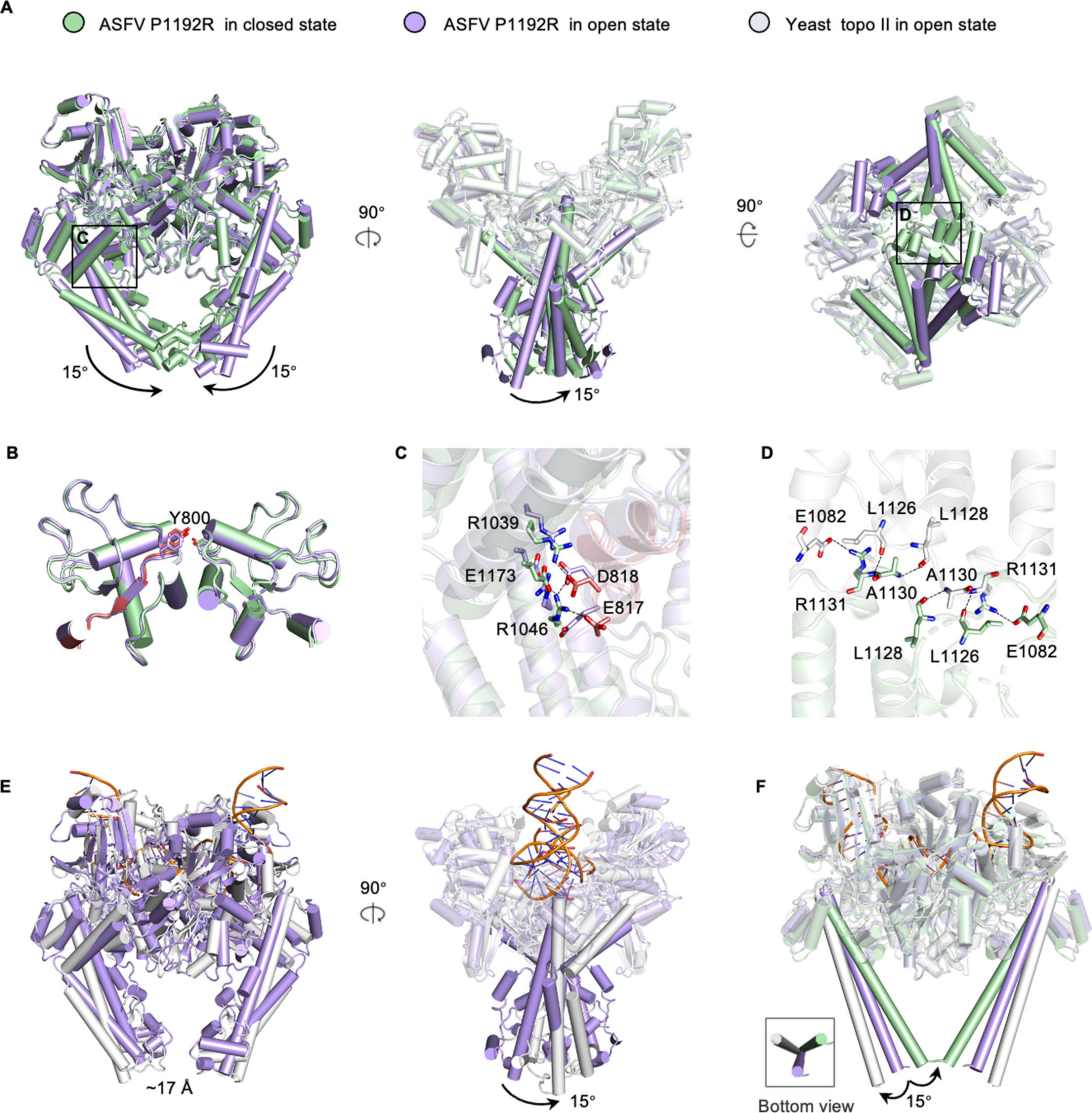
Conformational changes in the C-gate of ASFV P1192R. (**A**) Superimposition of the closed and open states of P1192R shows an ~15° rotating movement of the coiled-coil arm. The closed and open states are colored in green and purple, respectively. (**B**) Superimposition of the WHD domain of the closed and open states of P1192R. In the closed state, the distal linker from the active-site tyrosine to the coiled-coil arm is highlighted and colored in red. (**C**) Close-up view of the conserved salt-bridge networks in the closed and open states of P1192R. The E817-R1046 salt bridge is absent in the open state of P1192R. (**D**) Interactions at the C-gate interface of P1192R in the closed state. One protomer is in green, and the other is in gray. The interacting residues are labeled and shown as sticks. (**E**) Superimposition of the open states of P1192R and yeast topo II (PDB entry: 2RGR) shows that the coiled-coil arm rotates by ~15°. The yeast topo II is colored in gray, with the bound DNA in orange. (**F**) The closed state of P1192R is superimposed on the open states of P1192R and yeast topo II. The bottom left panel shows the rotational motion of the longest helix of the coiled-coil arm. For clarity, the other shorter helices of the coiled-coil arm are hidden in the structures.

To understand the structural changes in the C-gate region of P1192R, we performed superimposition analyses using the two P1192R structures and other available topo II structures from yeast and human (Fig. 3; Fig. S2). It shows that the conformations of the DNA cleave core for the two P1192R dimers are nearly identical, with a root-mean-square deviation (RMSD) value of 1.25 Å for all Cα. However, the coiled-coil arm of the closed state rotates inward by ~15° relative to that of the open state (Fig. 3A), thus leading to an enclosed C-gate interface stabilized via extensive intermolecular hydrogen bond and ionic interactions (Fig. 3D). A previous study showed that the rocking of the WHD upon DNA cleavage may control the motion of the coiled-coil arm by a distal linker and a conserved salt-bridge network, which in turn triggers a closed C-gate (33). In our work, the two states of P1192R display nearly identical WHD conformations (Fig. 3B), while differing in the salt-bridge network. Compared with the yeast topo II, P1192R contains an additional pair of E817-R1046 salt bridge, which is retained in the closed state while disrupted in the open state (Fig. 3C). Since no DNA substrates are present in the cryo-EM samples, we consider that the C-gate could adopt both closed and open conformations prior to reaction, and its status might be self-regulated through inter-domain interactions.

Comparison of the open states of P1192R and yeast topo II showed that the distance of the C-gate gap is nearly equal; however, the coiled-coil arm displays a 15° rotation between the two structures (Fig. 3E). Interestingly, we found that the longest α-helix in coiled-coil arm constitutes a tripod bracket with a bottom akin to an equilateral triangle when the closed state of P1192R was added for comparison (Fig. 3F). These observations suggest that the coiled-coil arm might be able to rotate at a certain angle in different directions. For the human topo II structure with an open C-gate, the coiled-coil arm displays a similar conformation to that observed in yeast topo II, but different from that of P1192R (Fig. S2).

### Structural differences in the DNA-gate of P1192R and yeast topo II

**Fig 4:**
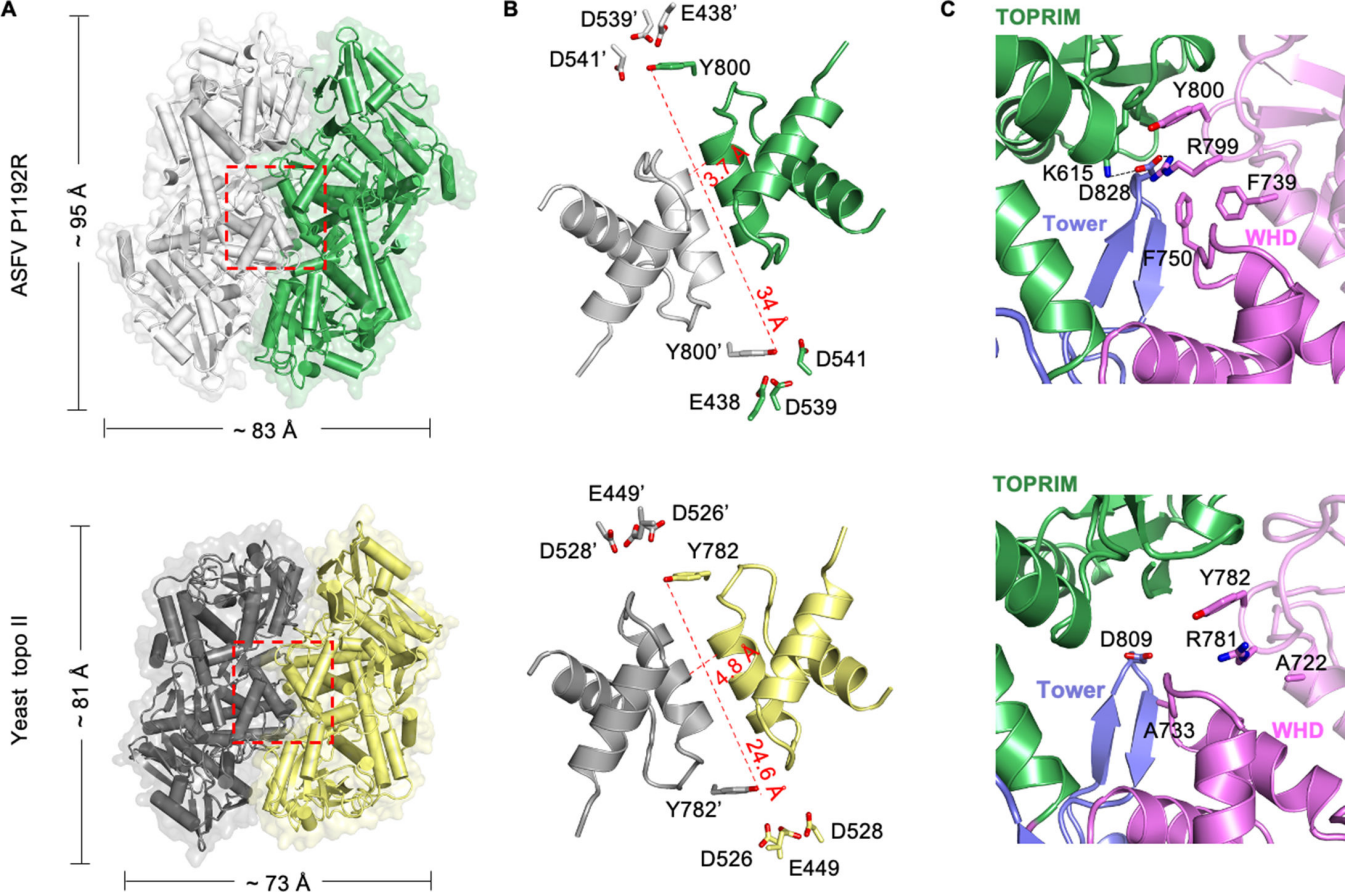
Structural comparison of the DNA-gate and active site of ASFV P1192R and yeast topo II. (**A**) Surface representation of the closed states of P1192R and yeast topo II [PDB entry: 4GFH (34)]. Each molecule has one protomer in gray and the other in color. The dimensions of the DNA-gate interface are determined by measuring the distance between the two Cα atoms of S657 and H892 (A647 and N988 in yeast topo II) and that of K427 and Q928 (K438 and G894 in yeast topo II). The DNA-gate between the WHD domains is denoted with the dotted box. For clarity, the ATPase domain of yeast topo II is hidden. (**B**) Comparison of the DNA-gate of P1192R and yeast topo II. For each structure, the catalytic tyrosines, the metal-binding residues, and the distance between two catalytic tyrosines are shown to illustrate the quaternary structural difference between these two structures. Residues belonging to the second monomer are flagged by a prime. (**C**) Comparison of the active site of P1192R and yeast topo II. The catalytic tyrosines and the residues involved in the active site interactions are labeled and shown as sticks. The coloring scheme for the domains is the same as in Fig. 2A. Domains from the second protomer are shown as semi-transparent.

The overall dimeric structure of P1192R is similar to those of other available type II topoisomerases from the prokaryotes and eukaryotes, with the highest similarity to yeast topo II (RMSD 3.3 Å). The structure deviation between the individual domains in the two structures is further diminished (RMSD ≤2.1 Å), and the WHD deviates the least (RMSD 0.9 Å) as the most conserved domain relative to others (Fig. S3A). Nonetheless, P1192R still exhibits an apparent discrepancy in the structural organization of the DNA-gate and the active site from that of yeast topo II (Fig. 4). First, the DNA-gate interface of P1192R has dimensions of roughly 95 Å high and 83 Å wide when viewed from the perspective of Fig. 4A, which is more extensive than that observed in yeast topo II. Moreover, in P1192R, the packing between the WHD domains is more compact, and the distance between the two catalytic tyrosines is farther compared with that of yeast topo II (Fig. 4B). Last, the active site of P1192R contains two pairs of salt bridge formed by three conserved charged residues (K615, R799, and D828), and a strong hydrophobic interaction involved in two phenylalanine residues on the WHD domain (F739 and F750) (Fig. 4C); these interactions together lock the subdomains from each subunit, giving rise to a close interface located in the center of DNA-gate. By contrast, in yeast topo II, both the salt bridge and hydrophobic interaction (the two phenylalanines were replaced with alanines) are absent in the active site (Fig. 4C), and it presents a relative open quaternary conformation in the center of the DNA-gate. Collectively, the structural difference of the DNA-gate between P1192R and yeast topo II might be related to the mechanism of enzyme action, such as the mode of the DNA binding and catalytic reaction.

### The unique structural features of P1192R are important for enzyme activity and protein folding

**Fig 5:**
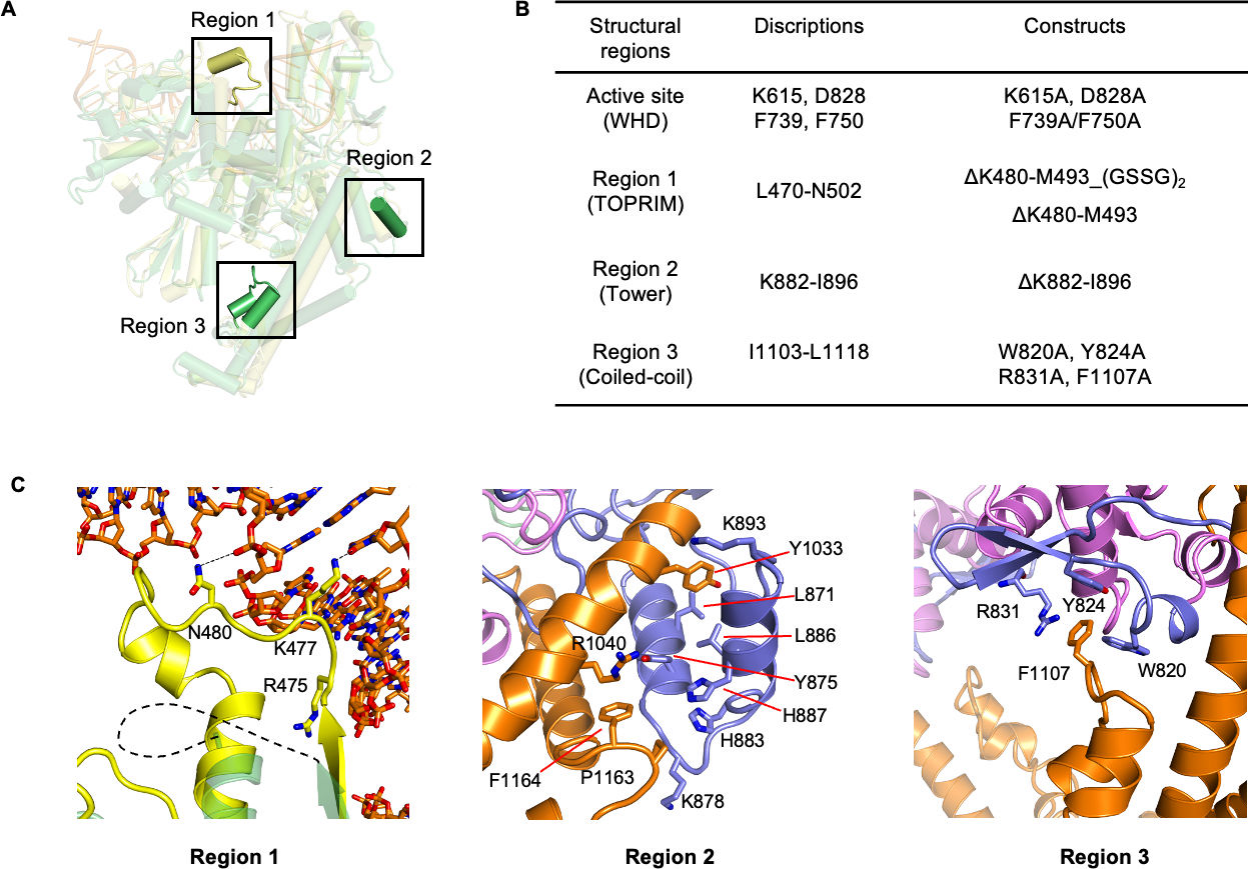
Functional regions in ASFV P1192R. (**A**) Superimposition of P1192R (green) and yeast topo II (yellow) monomers. The three different regions between P1192R and yeast topo II are highlighted with the black box. (**B**) A list of P1192R constructs used in this study, with full descriptions including the mutated regions, mutation site, and residue range. For region 1, two mutants were constructed with the unique insertion deleted directly or replaced with a (GSSG)_2_ linker. (**C**) Detailed interactions in the three regions of P1192R. Domains related to region 1 are colored as in (**A**), and those related to regions 2 and 3 are colored as in Fig. 2A. The key interacting residues in the three regions are labeled and shown as sticks.

Beyond the differences in quaternary conformation, structural variations also appear in the tertiary structure of P1192R and yeast topo II. Three distinct regions were identified between the two structures by superimposition analysis of the monomer (Fig. 5). Region 1, corresponding to residues L470-N502, is located in the TOPRIM domain and is flexible in both P1192R states, while it forms a short α-helix with a linked loop that involves DNA binding in yeast topo II (Fig. 5A and C). Compared with other type II topoisomerases, region 1 of P1192R harbors a unique insertion (K480-M493) likely participating in DNA binding (Fig. S4). Region 2 is an additionally inserted α-helix in the tower domain of P1192R, which makes extensive hydrophobic interactions with the coiled-coil arm. Region 3 is positioned at the coiled-coil domain and presents a pin-like structure formed by two short helices with a protruding loop, which harbors a phenylalanine that engages in a hydrophobic pocket formed by several bulk residues on the tower domain (Fig. 5A and C). In particular, region 3 is disorder in all reported topo II structures from yeast and human.

**Fig 6:**
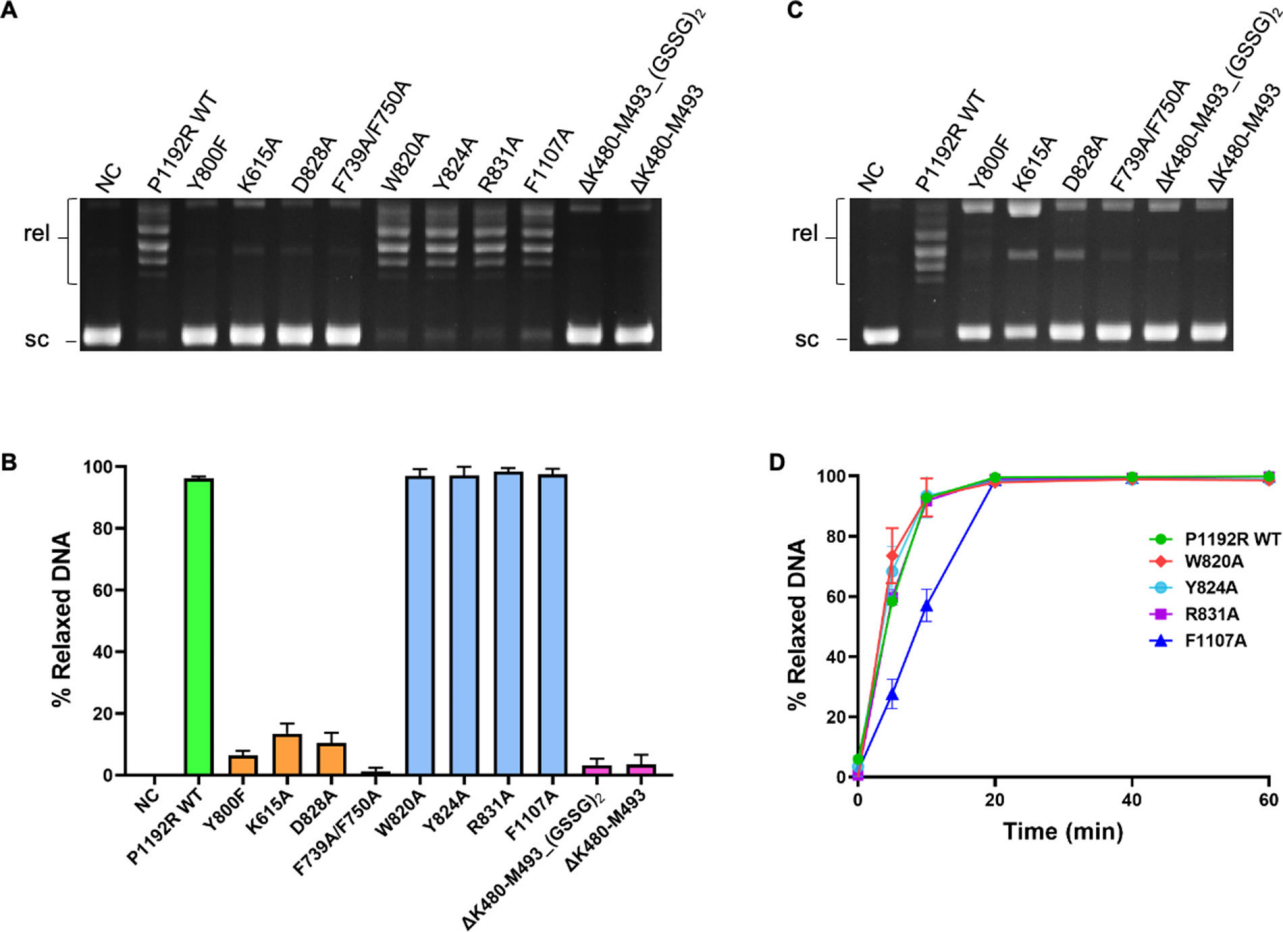
Relaxation activity of WT P1192R and mutants. (**A**) and (**B**) Determination of relaxation activity of WT P1192R and mutants. The pUC19 plasmids were incubated with 30 nM WT or indicated mutant protein for 40 min at 30°C. The incubation without enzyme was used as the negative control (NC). The gel of one representative experiment is shown. (**C**) Relaxation activity of WT P1192R, active site, and region 1 mutants at high enzyme concentration (120 nM). (**D**) Time course of relaxation activity of WT P1192R and region 3 mutants. The reaction was conducted as in Fig. 1. Supercoiled (sc) and relaxed (rel) topoisomers are indicated. For panels B and D, data are presented as mean values ± SD for three independent experiments.

To investigate the functions of the active site residues and the unique regions in the catalytic activity of P1192R, we designed four sets of mutations according to these regions (Fig. 5B), all aiming to disrupt the interactions between domain interfaces and the putative contacts with DNA substrate. The deletion mutant of region 2 (ΔK882-I896) had extremely low protein yield and was unstable, indicating the important role of region 2 in protein folding. All the other mutants were successfully prepared with high purity and subjected to *in vitro* relaxation assays. The results showed that mutations of the active site residues including K615, D828, F739/F750, and the deletion and substitution of the unique insertion of region 1 significantly reduced the relaxation activity compared with the WT P1192R (Fig. 6A and B). To further corroborate the results, we performed the relaxation activity assay at an extremely high enzyme concentration (120 nM), and the five P1192R variants still were unable to relax the supercoiled plasmids (Fig. 6C). Substitution of the residues (W820, Y824, R831, and F1107) involved in a hydrophobic pocket formed by region 3 and the tower domain retained the relaxation activity of P1192R (Fig. 6A and B); however, mutation of the core residue F1107 showed apparently lower activity than that of the WT protein at the early stage of reaction (Fig. 6D). Together, these data indicate that the P1192R-specific active site interactions are essential for the enzyme activity, and regions 1–3 are important for the function of P1192R.

## DISCUSSION

The topoisomerase family plays an essential role in DNA transcription and replication by changing the DNA topology. Structurally, topoisomerase is a delicate protein machine that implements complex catalytic reactions by flexible coordination of the individual domains. For type II topoisomerases, the G-segment binding, breaking, and T-segment transfer processes are regulated by timed sequential opening and closing of multiple domain interfaces (gates) induced by conformational changes. It has been believed that type II topoisomerase uses a two-gate mechanism for DNA strand transport, in which the DNA-gate first binds and cleaves the G-segment, then the T-segment passes through the transient break of the DNA-gate and escapes through an opening of the C-gate (35, 36). A previously proposed model relating the DNA and C-gate status in the context of type IIA topoisomerase reaction suggested the C-gate may adopt open or closed conformation prior to DNA binding (18). In our study, we observed that the P1192R simultaneously exists in two states in solution, with its C-gate closed or open (Fig. 2D), providing the first experimental evidence to support the previously proposed model. In addition, we found that the open state of the P1192R, yeast, and human topo II shared a similar opening distance (~17 Å) of the C-gate, while differing in the conformation of the coiled-coil arm (Fig. 3E; Fig. S2). Interestingly, although distinct in the rotation direction of the coiled-coil arm in these structures, its rotation angle is coincidentally identical (~15°) when using the closed state of P1192R as reference (Fig. 3E and F; Fig. S2). Collectively, these observations strongly suggest that the coiled-coil arm forming the C-gate is both flexible and stringent, which may not only contribute to the efficient transport of the T-segment from different directions but also preserve the accuracy of the strand passage reaction.

Despite the similar overall architecture, some diverse regions were observed in the structures of P1192R and other reported topo II. Compared with the yeast topo II, P1192R exhibits a more compact DNA-gate and a longer distance between the catalytic residues in the active site (Fig. 4A and B), which is probably due to the close inter- or intra-subunit contacts mediated by two pairs of salt bridge and a hydrophobic interaction (Fig. 4C). Our data showed that perturbation of these active site interactions abolishes the activity of P1192R (Fig. 6A to C), suggesting their critical role in the conformational stability of the DNA-gate that is required for the catalytic reaction. In addition, P1192R reveals a longer, positively charged groove stretched across the DNA-gate interface that likely accommodates DNA binding (Fig. S3B). These structural features may be related to the mode of DNA substrate binding and cleavage, such as the binding length and cleavage site. Besides the discrepancy in the quaternary organization of the DNA-gate, P1192R also features some new tertiary structural elements compared with the yeast topo II, which are important for enzyme activity (Fig. 5 and 6). For example, region 1 has an additional insertion that probably involves in DNA binding (Fig. S4) and explains why it displays a disordered configuration in the apo P1192R structures. The deletion and substitution of the insertion of region 1 almost eliminated the relaxation activity (Fig. 6A through C), indicating its essential role in the function of P1192R, and presumably due to its contribution to DNA binding or conformational stability of the TOPRIM domain in other functional states. Region 2 is an α-helix insertion that makes strong hydrophobic interactions with the coiled-coil arm (Fig. 5A and C). We did not obtain stable and sufficient mutant proteins of this region, probably because deletion of this α-helix impaired the protein folding. Region 3 is disordered and unmodeled in previous yeast and human topo II structures, while it is fully structured in the P1192R structure (Fig. 5A). Our structures revealed that this region engages in a hydrophobic pocket associated with a β-harpin of the tower domain adjacent to the active site (Fig. 5C). Mutation of the center hydrophobic residue F1107 in region 3 led to a reduced relaxation activity at the initial reaction stage (Fig. 6D), suggesting this region is also related to the enzyme activity of P1192R, possibly due to its contribution in the structural stability of the microenvironment around the active site.

P1192R plays a critical role in ASFV replication and is an attractive target for antiviral drug development. Previous investigations have tested the antiviral effects of several known antimicrobials and anticancer drugs targeting the type II topoisomerase on ASFV infection. The results showed that the tested drugs were either ineffective against ASFV or had poor inhibitory effects (9, 31, 37, 38). To probe the possible action mode of the type II topoisomerase drugs in ASFV P1192R, we superimposed the P1192R with the complex structures of human topo II–DNA–etoposide and *S. aureus* gyrase–DNA–ciprofloxacin (a fluoroquinolone drug) (25, 39), respectively (Fig. S5). In human topo II, the etoposide inserts into the base pairs at the DNA cleavage site and forms extensive interactions with a surrounding conserved PLRGKXL motif. However, this binding motif is changed in P1192R and exactly corresponds to the disorder region 1 (Fig. S5A). In *S. aureus* DNA gyrase, the binding mode of ciprofloxacin is similar to that of etoposide to human topo II. Interplay between the drug and the protein also involves a cluster of residues with identical spatial locations to region 1 of P1192R (Fig. S5B). Consequently, sequence and structural variations of this region probably hinder or reduce the binding of these drugs to P1192R, which may explain their low efficacy against ASFV infection.

Type II topoisomerase is found in all organisms of the three domains of life and several members of the virosphere. Owing to the diversity in the members and sequences, the evolutionary relationships among different type II topoisomerases are not clearly defined; especially, the phylogenetic position of the viral type II topoisomerases remains ambiguous. The previous phylogenetic tree analysis showed that viral and eukaryotic type II topoisomerases converged together; some viral members branch deep in the eukaryotes, while others branch between the bacteriophage T4 and eukaryotes (10, 40). Both the above scenarios suggest that the ancient horizontal gene transfer may occur between viruses and their hosts, but the direction of transfer is uncertain. A recent work performed in-depth phylogenetic analyses for type IIA topoisomerases strongly suggested the viral origin of eukaryotic type IIA topoisomerases (41). To discuss the possible phylogenies of type IIA topoisomerases from a structural perspective, we conducted structural comparisons of the ASFV P1192R structure and those of the representative eukaryotic and prokaryotic type IIA topoisomerases followed by average linkage clustering of the pairwise Z scores using DALI (42). In the structural dendrogram and similarity matrix, the eukaryotic and prokaryotic type II topoisomerases were divided into two separate branches (Fig. S6), suggesting their distinct origins. In addition, the P1192R formed a cluster with the yeast and human topo II, showing the closest similarity to the type IIA topoisomerase of eukaryotes, but distant from those of prokaryotes (Fig. S6A and B). Importantly, we found the eukaryotic type IIA topoisomerases nested within P1192R branch (Fig. S6A), indicating that the eukaryotic type IIA topoisomerases occurred later than the viral one. These results are largely consistent with the previous scenarios in which the eukaryotic type IIA topoisomerase was probably acquired from a viral ancestor of NCLDVs (41, 43). Regardless, the scenario of viral origin needs to be supported by further studies of structure, function, and evolution for the divergent type II topoisomerases.

## MATERIALS AND METHODS

### Expression and purification of ASFV P1192R

The DNA fragment encoding the ASFV P1192R (Gene ID: 59227094) was ligated into a modified yeast expression vector pPICZ-B fused with a PreScission protease cleavage site followed by a C-terminal GFP-His_10_ tag. The P1192R mutants were generated using the site-directed mutagenesis method. All constructs were transformed into the yeast *P. pastoris* (SMD1163H) for protein expression after verification by DNA sequencing. For protein purification, yeast cells were harvested and resuspended in a lysis buffer containing 20 mM Tris-HCl, pH 8.0, 1 M NaCl, 50 mM imidazole, 10% (vol/vol) glycerol, 0.1% Triton X-100, 1 mM PMSF, and DNase I. The cells were disrupted by high-pressure cell crusher (Union-Biotech) at 700 bars, followed by centrifugation at 30,000 *g* for 40 min at 4°C. The supernatant was collected and then loaded onto Ni-charged resin FF and washed with wash buffer containing 20 mM Tris-HCl, pH 8.0, 500 mM NaCl, 100 mM imidazole, and 10% (vol/vol) glycerol. The target protein was eluted with wash buffer supplemented with 500 mM imidazole and then digested with PreScission protease at 4°C overnight to remove the C-terminal GFP-His_10_ tag. Further purification was performed on a Superose 6 Increase 10/300 Gl size exclusion chromatography column (Cytiva) equilibrated with 20 mM Tris-HCl, pH 8.0, 500 mM NaCl, 5 mM DTT, and 5% (vol/vol) glycerol. Peak fractions containing P1192R protein were pooled and concentrated to 8 mg/mL, then divided into small aliquots, flash-frozen in liquid nitrogen, and stored at −80°C for further use.

### Relaxation activity assay

pUC19 plasmids were purified from the *E. coli* (DH5α) cells and used as the substrates. Reactions were conducted at 30°C in a 160  µL reaction volume containing 30 or 120 nM P1192R protein, 50 mM Tris-HCl, pH 8.0, 25 ng/µL of supercoiled pUC19, 2 mM ATP, 30 mM NaCl, 5 mM DTT, 5 mM MgCl_2_, and 5% (vol/vol) glycerol. An aliquot of 20 µL reaction mixture was withdrawn and quenched by adding 1% (wt/vol) SDS followed by digestion with 20 µg of proteinase K for 30 min at 37°C. The mixture was then added with 2 µL of loading buffer containing 0.08% (wt/vol) xylene cyanole F, 0.08% (wt/vol) bromophenol blue, 50 mM EDTA, pH 8.0, 60% (vol/vol) glycerol, and electrophoresed in a 1% (wt/vol) agarose gel with Tris-acetate-EDTA (TAE) buffer at room temperature for 40 min. Agarose gels were stained with 0.5 mg/mL GoldView in TAE buffer for 30 min and visualized under UV light. The DNA intensity quantification was performed using ImageJ software, and the percentage of relaxed pUC19 DNA was calculated by dividing the intensity of relaxed pUC19 DNA by that of the total pUC19 DNA.

### Cryo-EM sample preparation, data collection, and image processing

About 3 µl of purified P1192R protein at 0.3 mg/ml supplemented with 1 mM AMPPNP and 5 mM MgCl_2_ was applied to a glow-discharged Au 300 mesh R1.2/1.3 holey carbon grid (Quantifoil). After incubation for 5  s, the grids were blotted for 1.5  s at 100% humidity and 10°C and plunge-frozen in liquid ethane using Vitrobot Mark IV (FEI Thermo Fisher).

Cryo-EM data acquisition was performed at a CRYO ARM 300 electron microscope (JEOL, Japan) operating at 300 kV with a K3 direct electron detector (Gatan, USA). Cryo-EM movies were recorded in an automated fashion and in super-resolution mode with a super-resolution pixel size of 0.475 Å/pixel at defocus values ranging from −0.5 to −2.5 µm using Serial-EM software. Data were collected at a frame rate of 40 frames per second with a total electron dose of 40 e/Å2. Recorded movies were inputted into cryoSPARC for patch motion correction and CTF estimation (44); 37 bad images were removed and then subjected to particle picking using Topaz picking. In total, 914,196 particles were extracted from the micrographs with a box size of 300 pixels and subsequently subjected to 2D classification; 388,508 good particles were selected for ab-initio reconstruction and then heterogeneous refinement. Finally, two predominant classes containing 135,059 particles and 126,144 particles were selected for CTF refinement and nonuniform refinement (NU-refinement) with C2 symmetry imposed, yielding two reconstructions at an overall resolution of 3.16 Å and 3.13 Å, respectively.

### Model building and refinement

The structures were *de novo* built guided by bulky side chains to register the sequence. Cycles of model building and refinement were then carried out in Coot (45) and Phenix using phenix.real_space refine (46) with C2 symmetry imposed. The final models contain residues 415–470 and 502–1192. The quality of the models was analyzed with MolProbity in Phenix (47). Refinement statistics are summarized in Table S1.

## Data Availability

The atomic coordinates have been deposited in the Protein Data Bank under accession codes 8J9Y (P1192R in closed state) and 8J9Z (P1192R in open state). The cryo-EM maps have been deposited to Electron Microscopy Data Bank with accession codes EMD-36119 and EMD-36120.

## SUPPLEMENTAL MATERIAL

Supplemental Material mbio.01228-23-s0001.docx Fig. S1-S6 and Table S1.

ASM does not own the copyrights to Supplemental Material that may be linked to, or accessed through, an article. The authors have granted ASM a non-exclusive, world-wide license to publish the Supplemental Material files. Please contact the corresponding author directly for reuse.
