# Supplementary material for: Cryo-EM structures of African swine fever virus topoisomerase
Source: mBio. 2023 Aug 23;14(5):e01228-23. doi: 10.1128/mbio.01228-23 (PMC10653817; doi:10.1128/mbio.01228-23)
Supplement: Supplemental Material — Fig. S1-S6 and Table S1. [file mbio.01228-23-s0001.docx]

**Supplemental materials**

**Figure S1. Cryo-EM reconstruction of ASFV P1192R.** (A) Representative cryo-EM micrograph of ASFV P1192R. (B) Representative 2D class averages. (C) Flow chart of cryo-EM data processing. (D) and (E) Cryo-EM density maps colored by local resolution for P1192R in the closed and open state, respectively. (F) Fourier shell correlation (FSC) curve calculated using two independent half maps. Resolution was estimated using the FSC=0.143 cutoff. (G) Euler distribution of the refined particles.

**Figure S2. Superimposition of the C-gate open states of ASFV P1192R,** **yeast, and human topo II**. The C-gate conformation of P1192R differs from those observed in yeast and human topo II (PDB entry: 4FM9).

**Figure S3. Comparison of individual domains and electrostatic surface potential of ASFV P1192R, yeast, and human topo II.** (A) Superimposition of the individual domains of P1192R (green) and yeast topo II (yellow). The RMSD values are indicated using chain A of P1192R as reference. (B) Electrostatic surface representation of P1192R, yeast, and human topo II. The positive and negative electrostatic potentials are shown in blue and red, respectively. The positively charged groove highlighted with white dashed lines implies possible DNA binding site in P1192R. Scale bar: −3 kT/e in red to +3 kT/e in blue.

**Figure S4. Sequence alignment of the functional regions in ASFV P1192R and other type II topoisomerases.** The name of viruses, eukaryotes, and prokaryotes are colored in violet, green and blue, respectively. Residues in the active site are relatively conserved among different type II topoisomerases, while those in regions 2 and 3 labeled with the black box are largely variable. The K480-M493 insertion in region 1 is unique in ASFV P1192R. The residues selected for mutation are indicated, with the conserved residues colored in red. PBCV-1, Paramecium bursaria Chlorella Virus 1; APMV, Acanthamoeba polyphaga mimivirus; IIV-6, Invertebrate iridescent virus 6; BrMr, Brazilian marseillevirus; GVE3, Geobacillus virus E3.

**Figure S5. Superposition analysis of ASFV P1192R with the drug-bound topo II complex structures.** (A) and (B) P1192R in the closed state is superimposed on the complex structures of human topo II–DNA–etoposide and *S. aureus* gyrase–DNA–ciprofloxacin, respectively. Drugs are shown as sticks and colored yellow. DNA is shown in orange. Key residues involved in drug binding are indicated and the color for them correlates with Fig. 2A. Residues belonging to the second monomer are flagged by a prime. In ASFV P1192R, the conserved P^501^LRGKXL^507^ motif required for etoposide binding is replaced with the S^469^LGGVIM^475^ segment, which corresponds to the disorder region 1 denoted by dotted red line.

**Figure S6. Relationships among viral, eukaryotic, and prokaryotic type IIA topoisomerases.** (A) Structural dendrogram of ASFV P1192R and the representative type II topoisomerases from eukaryotes and prokaryotes. (B) Structural similarity matrix is based on the pairwise Z score comparisons calculated using DALI. The color scale indicates the corresponding Z scores. PDB identifiers are indicated next to the name of proteins.

**Table S1. Cryo-EM data collection, refinement and validation statistics**

|  | P1192R  Closed state  (PDB: 8J9Y)  (EMD-36119) | P1192R  Open state  (PDB: 8J9Z)  (EMD-36120) |
| --- | --- | --- |
| **Data collection and processing** |  |  |
| Magnification | 50,000 | 50,000 |
| Voltage (kV) | 300 | 300 |
| Electron exposure (e–/Å^2^) | 40 | 40 |
| Defocus range (μm) | -0.5 to -2.5 | -0.5 to -2.5 |
| Pixel size (Å) | 0.95 | 0.95 |
| Symmetry imposed | C2 | C2 |
| Initial particle images (no.) | 914,196 | 914,196 |
| Final particle images (no.) | 135,059 | 126,144 |
| Map resolution (Å)  FSC threshold | 3.16  0.143 | 3.13  0.143 |
| Map resolution range (Å) | 2.5-5 | 2.5-5 |
|  |  |  |
| **Refinement** |  |  |
| Initial model used | This study | This study |
| Model resolution (Å)  FSC threshold | 3.3  0.5 | 3.3  0.5 |
| Model resolution range (Å) | 3.1 | 3.1 |
| Map sharpening *B* factor (Å^2^) | -122.2 | -116.8 |
| Model composition  Non-hydrogen atoms  Protein residues | 12,154  1,494 | 12,154  1,494 |
| *B* factors (Å^2^ mean)  Protein | 58.8 | 66.1 |
| R.m.s. deviations  Bond lengths (Å)  Bond angles (°) | 0.002  0.498 | 0.002  0.411 |
| Validation  MolProbity score  Clashscore | 1.33  5.43 | 1.26  4.52 |
| Ramachandran plot  Favored (%)  Allowed (%)  Disallowed (%) | 97.9  2.1  0 | 97.9  2.1  0 |
